# Obstructive Sleep Apnea Increases the Risk of Perioperative Myocardial Infarction Following Off-Pump Coronary Artery Bypass Grafting

**DOI:** 10.3389/fcvm.2021.689795

**Published:** 2021-07-08

**Authors:** Kangjun Fan, Mingxin Gao, Wenyuan Yu, Hongli Liu, Liang Chen, Xiaohang Ding, Yang Yu

**Affiliations:** Department of Cardiac Surgery, Beijing Anzhen Hospital, Capital Medical University, Beijing, China

**Keywords:** obstructive sleep apnea, perioperative myocardial infarction, off-pump coronary artery bypass grafting, syntax score, high-sensitivity c-reactive protein

## Abstract

**Background and Aims:** The impact of obstructive sleep apnea (OSA) on perioperative myocardial infarction (PMI) following coronary artery bypass grafting (CABG) remains unclear. Off-pump CABG (OPCABG) has become a common practice for CABG in China. The present study investigated mainly the correlation between OSA and PMI following OPCABG.

**Methods:** In this prospective observational single-center study, consecutive eligible patients listed for elective OPCABG underwent cardiorespiratory polygraphy before surgery between January 2019 and June 2020. OSA was defined as an apnea-hypopnea index (AHI) ≥15 events/h. The primary end point was perioperative myocardial infarction (PMI) following OPCABG (type 5 MI).

**Results:** Patients with OSA accounted for 42.2% (62/147) of the cohort. Twenty-four patients (16.3%) met the protocol criteria for PMI: 17 (27.4%) in the OSA group and 7 (8.2%) in the non-OSA group (*P* = 0.002). Multivariate logistic regression analysis revealed that AHI (OR = 1.115, 95% CI 1.066 to 1.166, *P* < 0.001), high-sensitivity c-reactive protein (hs-CRP) (OR = 1.080, 95% CI 1.025 to 1.138, *P* = 0.004), and SYNTAX score (OR = 1.098, 95% CI 1.056 to 1.141, *P* < 0.001) were associated with PMI incidence. Furthermore, ROC analysis revealed that the AHI (AUC = 0.766, 95% CI 0.689 to 0.832, *P* < 0.001) and SYNTAX score (AUC = 0.789, 95% CI 0.715 to 0.852, *P* < 0.001) had predictive value for PMI. In addition, multiple linear regression analysis showed that the AHI was an independent influencing factor of hs-CRP (B = 0.176, 95% CI 0.090 to 0.263, *P* < 0.001) and the SYNTAX score (B = 0.553, 95% CI 0.397 to 0.709, *P* < 0.001).

**Conclusions:** OSA was independently associated with a higher incidence of PMI following OPCABG, and the formation of severe coronary atherosclerotic lesions aggravated by an enhanced inflammatory response might be the potential mechanism. The efficacy of CPAP treatment for improving prognosis after CABG remains to be further investigated.

## Introduction

Coronary artery bypass grafting (CABG) is the standard of care for patients with extensive coronary artery disease (CAD). Techniques to perform CABG without cardiopulmonary bypass (off-pump CABG, OPCABG) have emerged to reduce complications associated with cardiopulmonary bypass and manipulation of the aorta, especially for higher-risk patients who could benefit from short-term prognosis improvement (1). Despite decades of refinement, perioperative myocardial infarction (PMI) following CABG, with an incidence of 1.9–30%, remains a major predictor of increased cardiovascular mortality and healthcare resource utilization (2–4). Therefore, it is necessary to identify high-risk patients with PMI and initiate targeted treatment in advance.

Obstructive sleep apnea (OSA), characterized by recurrent episodes of partial or complete collapse of the upper airway during sleep, is highly prevalent in patients with CAD (5). OSA has been proven to be closely related to the degree of coronary atherosclerosis and myocardial infarction through activation of proinflammatory responses, oxidative stress, sympathetic excitation, platelets, or metabolic dysregulation (6). Compared with the general population, OSA, diagnosed by an apnea-hypopnea index (AHI) ≥15, has a significantly higher prevalence of ~50% in CABG patients (7). Considerable evidence has confirmed that OSA is strongly correlated with increased risks of new revascularization, typical angina, atrial fibrillation and prolonged length of hospital stay after CABG (7, 8); however, the impact of OSA on PMI following CABG is still unclear. Therefore, we investigated mainly the correlation between OSA and PMI following OPCABG (type 5 MI), which was defined by the fourth universal definition of MI (9).

## Materials and Methods

### Study Design

This prospective observational single-center study was conducted in Beijing Anzhen Hospital, Capital Medical University. We enrolled consecutive patients with coronary artery disease who were scheduled to undergo elective OPCABG and had not been diagnosed with OSA from January 2019 to June 2020. The study protocol, which was approved by the institutional review board (Approval No.: 2013025), was explained to all patients, all of whom signed informed consent.

### Patients

All participating patients, who were between 40 and 75 years old, underwent an overnight sleep study before OPCABG. The exclusion criteria were as follows: preoperative treatment with an intra-aortic balloon pump (IABP), pulmonary insufficiency (e.g., chronic obstructive pulmonary disease), central sleep apnea (≥50% central events or central AHI ≥10/h), body temperature >37.5°C, combination with other surgeries, on-pump CABG, and sleep study failure (patients without adequate signal recording).

### Overnight Sleep Study

Overnight sleep studies were performed after clinical stabilization (within 1 week after admission) by portable cardiorespiratory polygraphy (ApneaLink, Resmed, Australia), which might provide high diagnostic accuracy and offer an accessible alternative to full polysomnography. The reported sensitivity and specificity were 0.88 and 0.84, respectively (10). Nasal airflow, arterial oxygen saturation, thoracoabdominal movements, and snoring episodes were recorded. Sleep studies were scored according to the American Academy of Sleep Medicine 2007 guidelines (11). Apnea was defined as an absence of airflow lasting ≥10 s (obstructive if thoracoabdominal movement was present and central if thoracoabdominal movement was absent). Hypopnea was defined as a reduction in airflow of >30% for ≥10 s and associated with a decrease in arterial oxygen saturation >4%. AHI was defined as the number of apneas and hypopneas per hour of total recording time. The oxygen desaturation index (ODI) was the number of times that oxygen saturation decreased by >3% per hour. Recruited patients were categorized into OSA (AHI ≥ 15 events/h) and non-OSA (AHI <15 events/h) groups. A satisfactory polygraphy signal recorded for a minimum of 3 h was considered a valid test. All studies were double-scored manually by independent sleep technologists. Further, scoring was performed in cases of discrepancy by a senior consultant in sleep medicine. All OSA patients were recommended for further evaluation and consideration of continuous positive airway pressure (CPAP) therapy after discharge.

### OPCABG

Surgical indications were in accordance with the guidelines (12). The same experienced cardiac surgeon (Y.Y.) performed all surgeries. The quality of graft anastomosis met the criteria recommended by the Operation Quality Committee of Beijing Anzhen Hospital. Measurement of graft flow and pulsatility index (PI) was performed by placing a handheld flow probe (MediStim, Oslo, Norway) around the graft. After surgery, the patients were monitored in the intensive care unit (ICU) until ventilator removal, and vital signs were stable. Perioperative management was conducted according to the guidelines (13). Within 48 h after OPCABG, myocardial markers were routinely tested every 6 h, and electrocardiogram (ECG) and echocardiography were performed every 12 h.

### Outcome

The primary end point was CABG-related MI ≦48 h after the operation (type 5 MI), also known as perioperative myocardial infarction (PMI), defined by the fourth universal definition of MI [cardiac troponin (cTn) >10 times the 99th percentile upper reference limit or a rise of cTn values >20% if the baseline values were elevated or falling, in addition to ECG changes, angiographic findings or new regional wall motion abnormalities] (9). All relevant data were independently judged by two experienced doctors (L.H. L, D.X.H.), who were blinded to sleep study results.

### Statistical Analyses

For continuous variables, a normal distribution was assessed using the Kolmogorov-Smirnov test. Continuous variables are shown as the mean ± SD or median (first and third quartiles) and were compared by using Student's *t-*test or the Mann-Whitney *U*-test. Categorical variables are presented as percentages and were compared using the chi-square test. To analyze the association between PMI and other variables, multivariate logistic regression analysis was implemented (demographic characteristics, cardiovascular risk factors and variables with *p* < 0.1 in the univariate logistic regression were included in the model). Additionally, we assessed the predictive value of AHI, SYNTAX score and high-sensitivity c-reactive protein (hs-CRP) for PMI through receiver operating characteristic (ROC) curve analysis. The predictive values were evaluated with the area under the curve (AUC), and the difference in the AUC with statistical significance was assessed with the DeLong test. Furthermore, the correlation between AHI and hs-CRP or SYNTAX score was determined by multivariate linear regression analysis (the model included demographic characteristics, cardiovascular risk factors and variables with *p* < 0.1 in the univariate linear regression). All analyses were conducted with SPSS 24.0 (SPSS., Chicago, IL, USA) and MedCalc V.11.4 (MedCalc, Inc., Ostend, Belgium) assuming a 2-sided test with a 5% level of significance.

## Results

### Basic Characteristics and Overnight Sleep Study Results

**Figure 1:**
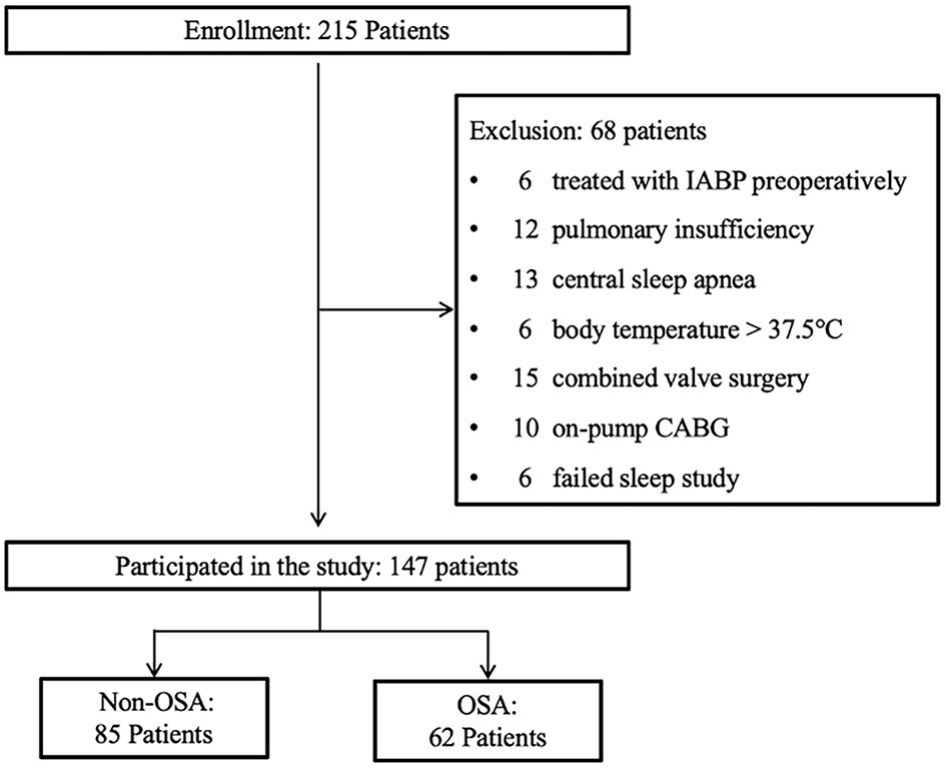
Study design flowchart. IABP, intra-aortic ballon pump; CABG, coronary artery bypass grafting; OSA, obstructive sleep apnea.

A total of 215 consecutive eligible patients scheduled for OPCABG were prospectively enrolled. After exclusion of 68 patients, 147 patients were included (Figure 1). The mean age of the patients recruited was 62.0 ± 8.9 years, and 74.8% were male. Patients with OSA accounted for 42.2% of the cohort and exhibited higher PCO_2_, percentage of BMI ≥ 28, hs-CRP levels and lower LVEF than the non-OSA group (Table 1). The median AHI level of patients with OSA was 26.2 (interquartile range, 14.3), and they had longer mean and maximum apnea times, higher oxygen desaturation indices, and lower lowest and mean arterial oxygen saturations than those without OSA (Table 2).

**Table 1:** Baseline clinical data for patients.

	**Total**	**Non-OSA**	**OSA**	***P*-value**
	**(*n* = 147)**	**(*n* = 85)**	**(*n* = 62)**	
**Demographic characteristics**				
Male, *n* (%)	110 (74.8%)	63 (74.1%)	47 (75.8%)	0.816
Age, y	62.0 ± 8.9	61.3 ± 9.3	62.9 ± 8.2	0.334
BMI, kg/m^2^	25.4 ± 3.4	25.5 ± 3.2	25.2 ± 3.7	0.602
BMI ≥ 28	31 (21.1%)	13 (15.3%)	18 (29.0%)	0.044
**Cardiovascular risk factors**, ***n*** **(%)**				
Hypertension	105 (71.4%)	58 (68.2%)	47 (75.8%)	0.316
Diabetes mellitus	62 (42.2%)	33 (38.8%)	29 (46.8%)	0.335
Smoker	74 (50.3%)	40 (47.1%)	34 (54.8%)	0.352
Alcohol abuse	76 (51.7%)	43 (50.6%)	33 (53.2%)	0.752
**Laboratory examination**				
PO_2_, mmHg	92.4 ± 21.1	94.8 ± 17.8	89.2 ± 24.6	0.129
PCO_2_, mmHg	36.2 ± 4.2	35.5 ± 3.3	37.2 ± 5.1	0.039
HDL, mmol/L	1.0 (0.9, 1.2)	1.0 (0.85, 1.2)	1.0 (0.9, 1.1)	0.311
LDL, mmol/L	2.2 (1.8, 3.0)	2.2 (1.8, 2.7)	2.4 (1.8, 3.2)	0.439
TG, mmol/L	1.3 (1.0, 1.9)	1.4 (1.1, 1.9)	1.3 (1.0, 1.9)	0.785
FPG, mmol/L	6.5 ± 2.2	6.4 ± 2.5	6.5 ± 1.8	0.685
Ccr, ml/min	92.9 ± 29.9	96.2 ± 31.3	88.4 ± 27.5	0.115
Hs-CRP, mg/L	1.6 (0.7, 5.3)	1.1 (0.3, 2.2)	2.2 (1.0, 8.2)	0.002
**Echocardiography data**				
LVEF, %	58.8 ± 9.3	60.3 ± 8.4	56.8 ± 10.2	0.030
LViDD, mm	49.3 ± 6.4	48.5 ± 6.6	50.3 ± 6.0	0.102
LAiD, mm	36.5 ± 3.9	36.9 ± 3.6	36.0 ± 4.2	0.132

*Values are mean (±SD), median (interquartile range), or No. (%). OSA, obstructive sleep apnea; BMI, body mass index; PO_2_, partial pressure of oxygen; PCO_2_, partial pressure of carbon dioxide; HDL, high density lipoprotein; LDL, lowdensity lipoprotein; TG, triglyceride; FPG, fasting plasma glucose; Ccr, Creatinine clearance rate; Hs-CRP, high-sensitivity c-reactive protein; LVEF, left ventricular ejection fraction; LViDD, left ventricular internal end diastolic diameter; LAiD, left atrium internal diameter*.

**Table 2:** Sleep monitoring findings of patients.

	**Total**	**Non-OSA**	**OSA**	***P*-value**
	**(*n* = 147)**	**(*n* = 85)**	**(*n* = 62)**	
AHI, events per hour	12.1 (6.3, 24.6)	7.0 (3.3, 10.0)	26.2 (20.5, 34.8)	<0.001
Mean apnea time, s	21.0 (15.5, 27.2)	17.0 (12.3, 24)	24.0 (19.8, 30.9)	<0.001
Maximum apnea time, s	32.0 (19.3, 47.1)	23.5 (13, 36.3)	43 (31.8, 63.5)	<0.001
Lowest SaO_2_, %	84.2 ± 6.2	86.8 ± 3.8	80.6 ± 7.1	<0.001
Mean SaO_2_, %	94.0 ± 2.5	94.6 ± 2.3	93.2 ± 2.5	0.013
ODI	11.7 (4.9, 23.3)	6.5 (3.2, 9.6)	24.1 (18.4, 34.8)	<0.001
ESS score	9.0 (6.0, 12.0)	9.0 (6.0, 12.0)	9.0 (6.0, 13.0)	0.284

*Values are median (interquartile range), mean (±SD). OSA, obstructive sleep apnea; AHI, apnea hypopnea index; SaO_2_, arterial oxygen saturation; ODI, oxygen desaturation index; ESS, epworth sleepiness scale*.

### Angiographic and Surgical Characteristics

Angiographic and OPCABG surgical characteristics are shown in Table 3. The mean SYNTAX score of all patients was 52.6 ± 15.6, and in the OSA group, it was significantly higher than that in the non-OSA group (*P* < 0.001). The number of target vessels, distal anastomoses/patient, graft type, and assessment of grafts did not differ between the OSA and non-OSA groups.

**Table 3:** Angiographic and surgical characteristics.

	**Total**	**Non-OSA**	**OSA**	***P*-value**
	**(*n* = 147)**	**(*n* = 85)**	**(*n* = 62)**	
**Diseased vessel**, ***n*** **(%)**				
Left main stem disease	33 (22.4%)	16 (18.8%)	17 (27.4%)	0.217
Three-vessel disease	122 (83.6%)	72 (85.7%)	50 (80.6%)	0.414
**Target vessel**				
SYNTAX score	52.6 ± 15.6	47.7 ± 12.1	59.4 ± 17.4	<0.001
LAD, *n* (%)	147 (100%)	85 (100%)	62 (100%)	
Diag, *n* (%)	141 (95.9%)	80 (94.1%)	61 (98.4%)	0.384
OM, *n* (%)	108 (73.5%)	63 (74.1%)	45 (72.6%)	0.835
RCA, *n* (%)	30 (20.4%)	16 (18.8%)	14 (22.6%)	0.577
PDA, *n* (%)	111 (75.5%)	69 (81.2%)	42 (67.7%)	0.062
PLA, *n* (%)	76 (51.7%)	39 (45.9%)	37 (59.7%)	0.098
Distal anastomoses/patient	4.2 ± 0.5	4.2 ± 0.5	4.2 ± 0.5	0.842
**Worst target artery quality**				
Good	66 (44.9%)	42 (49.4%)	24 (38.7%)	0.044
Fair	40 (27.2%)	26 (30.6%)	14 (22.6%)	
Poor	41 (27.9%)	17 (20.0%)	24 (38.7%)	
**Graft type**, ***n*** **(%)**				
LIMA graft + SVG	123 (83.7%)	72 (84.7%)	51 (82.3%)	0.692
**Assessment of graft**				
Q_mean_ of all grafts, ml/min	24 (15, 40)	24.5 (16, 40)	24 (14, 40)	0.565
PI_mean_ of all grafts	2.2 (1.7, 2.8)	2.1 (1.7, 2.7)	2.3 (1.8, 2.8)	0.123
Q_mean_ of arterial grafts, ml/min	24 (16, 42)	23 (15.3, 36.5)	30 (17, 43)	0.081
PI_mean_ of arterial grafts	2.1 (1.7, 2.7)	2 (1.6, 2.7)	2.3 (1.7, 2.8)	0.355
Q_mean_ of SVGs, ml/min	24 (15, 40)	25.5 (16, 40.8)	23 (13, 39)	0.144
PI_mean_ of SVGs	2.2 (1.7, 2.8)	2.1 (1.7, 2.8)	2.3 (1.8, 2.8)	0.206

*Values are mean (±SD), media n (interquartile range), or No. (%). OSA, obstructive sleep apnea; LAD, left anterior descending branch; Diag, diagonal branch; OM, obtuse marginal branch; RCA, right coronary artery; PDA, posterior descending artery; PLA, posterior left ventricular artery; LIMA, left internal mammary artery; SVG, saphenous vein graft; Q_mean_, mean flow; PI, pulsatility index*.

### Primary End Point

**Figure 2:**
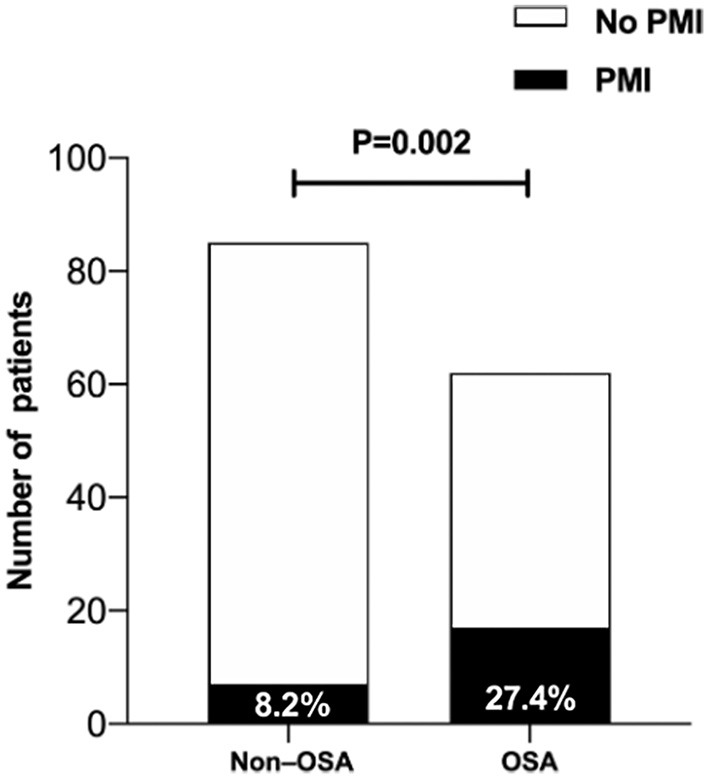
The proportion of patients with PMI in the moderate-severe OSA group was significantly higher than in the non-OSA group. PMI, periprocedural myocardial infarction; OSA, obstructive sleep apnea.

Twenty-four patients (16.3%) met the protocol criteria for PMI: 17 (27.4%) in the OSA group and 7 (8.2%) in the non-OSA group (*P* = 0.002) (Figure 2). Among the 24 cases, there were 6, 6 and 12 cases of PMI of the anterior, lateral and inferior wall, respectively, and there was no significant difference between the OSA group and non-OSA group. Extracorporeal membrane oxygenation (ECMO) and IABP were used in 1 and 6 patients, respectively; two cases died of PMI. Univariate logistic regression analysis revealed that AHI (OR = 1.088, 95% CI 1.050 to 1.127, *P* < 0.001), hs-CRP (OR = 1.075; 95% CI 1.027 to 1.125, *P* = 0.002) and SYNTAX score (OR = 1.086, 95% CI 1.049 to 1.123, *P* < 0.001) were strongly associated with an increased risk of developing PMI (Table 4). After adjustments for sex, age, BMI, hypertension, diabetes mellitus, smoking status, alcohol abuse, and left atrial internal diameter through multivariate logistic regression analysis, the correlation persisted (OR = 1.115, 95% CI 1.066 to 1.166, *P* < 0.001; OR = 1.080, 95% CI 1.025 to 1.138, *P* = 0.004; OR = 1.098, 95% CI 1.056 to 1.141, *P* < 0.001, respectively) (Table 5). As the ROC analysis revealed, the AHI (AUC = 0.766, 95% CI 0.689 to 0.832, *P* < 0.001) and SYNTAX score (AUC = 0.789, 95% CI 0.715 to 0.852, *P* < 0.001) had predictive value for PMI (Table 6).

**Table 4:** Univariate logistic regression analysis for the correlation between various indicators and PMI.

**Covariate**	**Odds ratio (95% CI)**	***P*-value**
**Demographic characteristics and cardiovascular risk factors**		
Male	1.336 (0.461 to 3.874)	0.593
Age	0.989 (0.949 to 1.030)	0.593
BMI	0.913 (0.802 to 1.040)	0.171
Hypertension	0.966 (0.369 to 2.531)	0.944
Diabetes mellitus	0.509 (0.197 to 1.315)	0.163
Smoker	1.201 (0.500 to 2.888)	0.682
Alcohol abuse	1.694 (0.690 to 4.162)	0.250
**Sleep monitoring findings**		
AHI	1.088 (1.050 to 1.127)	<0.001
Mean apnea time	1.065 (1.012 to 1.119)	0.015
Maximum apnea time	1.036 (1.018 to 1.055)	<0.001
Lowest SaO_2_	0.891 (0.831 to 0.956)	0.001
Mean SaO_2_	0.728 (0.557 to 0.953)	0.021
≥3% ODI	1.066 (1.034 to 1.100)	<0.001
ESS score	1.128 (1.004 to 1.266)	0.042
**Other variables**		
Hs-CRP	1.075 (1.027 to 1.125)	0.002
LAiD	1.111 (0.998 to 1.237)	0.054
SYNTAX score	1.086 (1.049 to 1.123)	<0.001

*PMI, periprocedural myocardial infarction; CI, confidence interval; BMI, body mass index; AHI, apnea hypopnea index; SaO_2_, arterial oxygen saturation; ODI, oxygen desaturation index; ESS, Epworth Sleepiness Scale; Hs-CRP, high-sensitivity c-reactive protein; LAiD, left atrium internal diameter*.

**Table 5:** Multivariate logistic regression analysis for the correlation between various indicators and PMI.

**Covariate**	**Mode A**		**Mode B**		**Mode C**	
	**Odds ratio** **(95% CI)**	***P*-value**	**Odds ratio** **(95% CI)**	***P*-value**	**Odds ratio** **(95% CI)**	***P*-value**
Male	2.244 (0.448 to 11.234)	0.325	1.388 (0.377 to 5.103)	0.622	0.852 (0.183 to 3.963)	0.838
Age	0.954 (0.907 to 1.003)	0.064	0.973 (0.931 to 1.017)	0.223	0.967 (0.921 to 1.014)	0.166
BMI	0.817 (0.670 to 0.995)	0.044	0.906 (0.793 to 1.036)	0.150	0.915 (0.790 to 1.061)	0.239
Hypertension	0.659 (0.187 to 2.321)	0.516	0.779 (0.256 to 2.369)	0.660	0.896 (0.262 to 3.056)	0.860
Diabetes mellitus	0.288 (0.081 to 1.017)	0.053	0.467 (0.163 to 1.338)	0.156	0.294 (0.085 to 1.019)	0.054
Smoker	0.825 (0.242 to 2.814)	0.759	1.325 (0.451 to 3.888)	0.609	1.339 (0.397 to 4.514)	0.637
Alcohol abuse	2.370 (0.699 to 8.034)	0.166	1.914 (0.710 to 5.160)	0.199	2.604 (0.809 to 8.376)	0.108
LAiD	1.185 (1.030 to 1.364)	0.018	1.135 (1.006 to 1.281)	0.040	1.173 (1.021 to 1.347)	0.024
AHI	1.115 (1.066 to 1.166)	<0.001				
Hs-CRP			1.080 (1.025 to 1.138)	0.004		
SYNTAX score					1.098 (1.056 to 1.141)	<0.001

*PMI, periprocedural myocardial infarction; CI, confidence interval; BMI, body mass index; LAiD, left atrium internal diameter; AHI, apnea hypopnea index; Hs-CRP, high-sensitivity c-reactive protein*.

**Table 6:** Predictive value of AHI, hs-CRP and SYNTAX score for PMI.

	**AUC**	**95% CI**	***P* for AUC**	***P* for δAUC**	**Cut-off value**	**Sensitivity**	**Specificity**	**PPV**	**NPV**
AHI	0.766	0.689 to 0.832	<0.001	0.741	25.8	70.83%	87.80%	53.1%	93.9%
SYNTAX score	0.789	0.715 to 0.852	<0.001		63.0	66.67%	82.93%	43.2%	92.7%
Hs-CRP	0.638	0.555 to 0.716	0.053						

*AHI, apnea hypopnea index; Hs-CRP, high-sensitivity c-reactive protein; PMI, periprocedural myocardial infarction; AUC, area under curve by receiver-operating characteristic curve analysis; δAUC, difference of AUC; CI, confidence interval; PPV, positive predictive values; NPV, negative predictive values*.

### AHI Was Strongly Correlated With hs-CRP and SYNTAX Score

As demonstrated in Table 7, multiple linear regression analysis showed that AHI was independently associated with hs-CRP (B = 0.176, 95% CI 0.090 to 0.263, *P* < 0.001) and SYNTAX score (B = 0.553, 95% CI 0.397 to 0.709, *P* < 0.001).

**Table 7:** Univariate and multivariate linear regression analysis for the independent influencing factors of hs-CRP level and SYNTAX score.

**Covariate**	**Dependent variable: Hs-CRP**				**Dependent variable: SYNTAX score**			
	**Univariate**		**Multivariate**		**Univariate**		**Multivariate**	
	**B** **(95% CI)**	***P-*value**	**B** **(95% CI)**	***P-*value**	**B** **(95% CI)**	***P-*value**	**B** **(95% CI)**	***P-*value**
Male	−0.396 (−3.362 to 2.569)	0.792	0.075 (−2.978 to 3.128)	0.961	4.349 (−1.501 to 10.199)	0.144	4.733 (−0.813 to 10.280)	0.094
Age	0.090 (−0.037 to 0.217)	0.163	0.031 (−0.117 to 0.178)	0.681	0.137 (−0.116 to 0.390)	0.288	−0.007 (−0.287 to 0.272)	0.959
BMI	−0.089 (−0.459 to 0.281)	0.636	−0.045 (−0.414 to 0.324)	0.809	−0.442 (−1.174 to 0.291)	0.235	−0.278 (−0.966 to 0.410)	0.426
Hypertension	1.872 (−0.961 to 4.705)	0.194	1.549 (−1.145 to 4.243)	0.257	0.964 (−4.695 to 6.624)	0.737	0.074 (−4.839 to 4.986)	0.976
Diabetes mellitus	−0.218 (−2.825 to 2.388)	0.869	−1.053 (−3.490 to 1.383)	0.394	2.523 (−2.639 to 7.686)	0.336	−0.159 (−4.547 to 4.230)	0.943
Smoker	−0.242 (−2.817 to 2.332)	0.853	−0.070 (−2.697 to 2.556)	0.958	−0.117 (−5.233 to 4.998)	0.964	−2.987 (−7.772 to 1.798)	0.219
Alcohol abuse	0.135 (−2.441 to 2.711)	0.918	0.807 (−1.602 to 3.216)	0.509	−0.518 (−5.636 to 4.600)	0.842	−0.046 (−4.432 to 4.339)	0.983
LDL	2.018 (0.756 to 3.280)	0.002	2.001 (0.765 to 3.237)	0.002				
TG					−2.195 (−4.323 to −0.067)	0.043	−2.054 (−4.070 to −0.037)	0.046
Ccr	−0.040 (−0.083 to 0.003)	0.066	−0.013 (−0.067 to 0.041)	0.632	−0.076 (−0.161 to 0.009)	0.078	0.015 (−0.083 to 0.113)	0.762
LVEF	−0.132 (−0.269 to 0.005)	0.058	−0.115 (−0.254 to 0.023)	0.102	−0.525 (−0.786 to −0.263)	0.000	−0.335 (−0.638 to −0.032)	0.030
LViDD					0.490 (0.095 to 0.885)	0.015	0.029 (−0.423 to 0.480)	0.901
AHI	0.205 (0.121 to 0.290)	<0.001	0.176 (0.090 to 0.263)	<0.001	0.588 (0.435 to 0.740)	<0.001	0.553 (0.397 to 0.709)	<0.001

*Hs-CRP, high-sensitivity c-reactive protein; CI, confidence interval; BMI, body mass index; LDL, lowdensity lipoprotein; TG, triglyceride; Ccr, Creatinine clearance rate; LVEF, left ventricular ejection fraction; LViDD, left ventricular internal end diastolic diameter; AHI, apnea hypopnea index*.

## Discussion

To the best of our knowledge, this is the first study to evaluate the impact of OSA on PMI following OPCABG. In this prospective cohort of 147 patients who underwent OPCABG, the prevalence of OSA was 42.2%, and compared with in non-OSA patients, PMI was more likely to occur in patients with OSA. AHI, hs-CRP, and SYNTAX score were independent risk factors for PMI. In regard to identifying patients at high risk of having PMI, the AHI, and SYNTAX score could provide a superior discriminatory ability to that of hs-CRP. In addition, AHI was associated with changes in hs-CRP and SYNTAX score.

Along with the in-depth understanding of the pathogenesis and prognostic significance of PMI following CABG, different definitions have emerged, and the incidence of PMI ranges from 1.9 to 30% depending on different diagnostic criteria and patient populations (2, 3). The Fourth Universal Definition of Myocardial Infarction (2018) (9), applied in this study, remains the accepted standard in contemporary clinical practice. In addition, the diagnosis of PMI in several large randomized controlled trials was based on the creatine kinase-myocardial band (CK-MB) (14), but recently, the ESC Study Group on Cardiac Biomarkers of the Association for Acute Cardiovascular Care suggested that CK-MB be eliminated from the menu of biomarkers available since it was less sensitive and clinically instructive than cTn (15). We diagnosed patients based on cTn combined with other presentations and found that PMI following OPCABG occurred in 16.3% of patients with severe coronary lesions (mean SYNTAX score was 52.6).

An analysis from the EXCEL (Evaluation of XIENCE vs. Coronary Artery Bypass Surgery for Effectiveness of Left Main Revascularization) trial showed that PMI was associated with cardiovascular death and all-cause death at 3 years (16). In a Korean registry of 3,183 patients who underwent CABG, the presence of PMI was associated with increased risks of major cardiovascular events at the 5-year follow-up regardless of the PMI definition (2). PMI can be ascribed to multiple causes. In the EXCEL trial, age, chronic obstructive pulmonary disease, and aortic cross-clamp duration were independent predictors of PMI following CABG (16). Other well-established risk factors for PMI include left main coronary artery disease, recent MI, unstable angina, emergent operations, poor target-artery quality, inadequate revascularization, and prolonged bypass duration (17). However, few studies have focused on OPCABG. Our findings extend those of previous studies by proposing that OSA increases the risk of PMI in patients who underwent OPCABG. Of note, we found no significant correlation between the incidence of PMI and the measurement index of graft (flow and PI), the reason for this result might be that all indices met the quality control standard (flow>15/min and PI < 5.0) (18).

OSA has been advocated as a new emerging contributor to the development of CAD by guidelines and expert consensus (19, 20), and intravascular ultrasound has indicated that patients with OSA present a larger total atheroma volume in the coronary artery (21). The SYNTAX (SYNergy between PCI with TAXUS and Cardiac Surgery) score was created, more than a decade ago, to provide an angiographic tool grading the complexity of coronary artery disease in patient with left main or multivessel disease, and it relates to CAD outcomes (22). Evidence has revealed that OSA significantly correlates with the SYNTAX score (23). Our study supported this finding and further demonstrated that both the AHI and SYNTAX score could identify patients at high risk for developing PMI following OPCABG; however, the accuracy is still moderate, and their performance should be further explored.

Intermittent hypoxia (IH), sleep fragmentation, and intrathoracic pressure swings in OSA appear to promote the development of cardiovascular disease, and inflammation is proposed as an intermediate mechanism linking OSA with the severity of coronary lesions (6). IH appears to facilitate atheromatous lesion formation by promoting systemic inflammation, partially *via* activation of the transcription factor NF-κB (24). The most studied biomarker in this context is hs-CRP, which has been demonstrated to participate actively in atherosclerosis *via* the induction and enhanced expression of adhesion molecules (25). Our findings were consistent with previous literature: hs-CRP levels were strongly associated with the severity of OSA. We added further evidence that the hs-CRP level was an independent risk factor for PMI following OPCABG; however, its limited predictive value for PMI might be due to its susceptibility to multiple interference factors.

In addition, evidence has confirmed that patients with OSA show reduced fibrinolytic capacity (26) and increased platelet activation and aggregation (27), all of which may contribute to PMI *via* thrombotic events of grafts or native vessels after CABG.

CPAP remains the standard of care for patients with OSA, and evidence has revealed that it is correlated with a reduced inflammatory response (28) and coagulability (29), as well as improvement in early signs of atherosclerosis (30), suggesting that CPAP may improve the prognosis of coronary revascularization. Wu et al. (31) reported that untreated moderate-to-severe OSA was independently correlated with a significantly increased risk of repeat revascularization after percutaneous coronary intervention (PCI). Cassar et al. (32) found that screening for and treating OSA (CPAP) in patients who underwent PCI might result in decreased cardiac death. However, we know little about the role of CPAP in reducing the incidence of complications after CABG. In consideration of our previous clinical practice, we speculate that patient compliance and timing of CPAP treatment after CABG may be the key to curative effects. Additional trials evaluating the effects of CPAP in patients with OSA who underwent CABG are warranted.

This study has several limitations. First, this was a single-center study with a limited sample size, and the results need to be validated through multicenter, large-sample studies. Follow-up of the cohort is needed. Second, compared with polysomnography, portable cardiorespiratory polygraphy may underestimate the severity of OSA, but it is safe and simple to identify OSA in a high-risk patient population (11). Finally, mechanical ventilation treatment after surgery might weaken the short-term effect of OSA to a certain extent.

## Conclusions

This prospective cohort study demonstrated for the first time that OSA was independently associated with a higher incidence of PMI following OPCABG, and the formation of severe coronary atherosclerotic lesions aggravated by an enhanced inflammatory response might be the potential mechanism. The efficacy of CPAP treatment for improving prognosis after CABG remains to be further explored.

## Data Availability Statement

The raw data supporting the conclusions of this article will be made available by the authors, without undue reservation.

## Ethics Statement

This study was approved by the Institutional Review Board of Beijing Anzhen Hospital of Capital Medical University (Approval No: 2013025). The patients/participants provided their written informed consent to participate in this study.

## Author Contributions

KF, MG, and YY: study concept and design. KF, MG, WY, HL, LC, and XD: acquisition, analysis, or interpretation of data. KF and MG: drafting of the manuscript. WY and YY: obtained funding. All authors: critical revision of the manuscript for important intellectual content, read and approved the final manuscript.

## Conflict of Interest

The authors declare that the research was conducted in the absence of any commercial or financial relationships that could be construed as a potential conflict of interest.
